# Evaluation of a skills-based peer-led art therapy online-group for people with emotion dysregulation

**DOI:** 10.1186/s40479-022-00203-y

**Published:** 2022-11-30

**Authors:** Mahlie Jewell, Rachel C Bailey, Renae L Curran, Brin F.S. Grenyer

**Affiliations:** 1 School of Psychology and Illawarra Health and Medical Research Institute, University of Wollongong, 2522 Sydney, NSW Australia; 2 Western Sydney University, Sydney, Australia

**Keywords:** Borderline personality disorder, Online art therapy, Peer, Intentional peer support, Lived experience, Group therapy, Emotional dysregulation, Qualitative research, Psychotherapy

## Abstract

**Objective:**

We developed and piloted a novel art-based online skills program led by a peer mental health professional with lived experience of complex mental health, including Borderline Personality Disorder (BPD). Key challenges of living with BPD and emotion dysregulation were addressed through artmaking informed by a dialectical framework and skills, to evaluate acceptability and efficacy.

**Method:**

A structured, manualised 2-hour weekly arts-based skills program was piloted for people with BPD over 18 weeks. Evaluation included both quantitative and qualitative measures at commencement and completion.

**Results:**

Thirty-eight participants enrolled in the program (89.5% identified she/her pronouns, average age 33.6 years), and 31 completed (82% retention). Multilevel modelling analysis of the primary outcome variable Difficulties in Emotion Regulation Scale (DERS) demonstrated a large improvement over time (effect size *Cohen’s d* = 1.77). Qualitative thematic analysis found participants had improved capacity to regulate emotions and tolerate distress, improved connection with others, enhanced understanding of the self, and higher hope for living well. We found that artmaking facilitated processes and helped the expression of difficult emotions, symbolise challenging relationships, and facilitate greater self-understanding. Participants reported high levels of satisfaction, and 77.4% reported that the program had increased wellbeing.

**Conclusion:**

This novel artmaking program for emotion dysregulation and BPD was acceptable and potentially effective. Peer facilitation using arts-based skills is a modality of therapy for further investigation.

## Background

Personality disorders are a highly prevalent mental health condition, with the global prevalence of any personality disorder estimated at 7.8% [1]. Borderline Personality Disorder (BPD) in particular is characterised by difficulties regulating emotions, complex experiences of identity, impulsivity and psychosocial difficulties often resulting in self-harming or suicidal behaviours [2]. BPD is highly prevalent in mental health settings, with over 20% of mental health Emergency Department presentations and inpatient admissions meeting criteria for the diagnosis [3]. Psychological therapies have been developed and evaluated, and are now recognised as the treatment of choice for people living with BPD [4–6]. However, these therapies are often long-term in duration, resulting in well documented difficulties in practical application in time and resource limited health settings [7–9]. Additional access-related difficulties can also present barriers to engagement, including cost to the person with lived experience in the private setting, medicalised and non-inclusive models of care, and resource allocation being centred in metro areas with limited opportunities for rural and remote communities. Therefore, innovative approaches are needed to facilitate treatment accessibility and enhance engagement. Recent innovations include stepped-care approaches [10], integration of lived experience peer workers [11], and briefer forms of evidence-based therapies [12].

One example of a well-studied therapy is based on dialectical principles (Dialectical Behaviour Therapy; DBT). The traditional model consists of group and individual therapy, with access to phone coaching as needed, often for at least 12 months duration [13]. Recent studies have evaluated the effectiveness of brief therapy [12, 14, 15] or skills group only adaptations [16–19] with good outcomes in reducing the emotional and behavioural dysregulation in acute phase of illness (e.g. suicidal behaviour and self-harm). However, traditional psychological models of talk therapy for BPD with a primary focus on improving emotion regulation may not address complex experiences of identity inherent in the diagnosis. This is particularly salient in brief or adapted formats. Therefore complex difficulties associated with identity may require more targeted treatment [20–22]. Research shows that complex experiences of identity and chronic emptiness are associated with poor vocational outcomes for people living with BPD [23–25], and is core to the development and treatment of the diagnosis [26]. Therefore, dialectically-informed approaches may benefit from incorporating components designed to enhance identity formation and facilitate connection to a stronger sense of self [27]. This may include developing an enhanced connection to identified values, working through and changing negative self-narrative and self-stigma, expression of self, experience of worth, appreciation of strengths, uniqueness, and creativity, amongst other important identity considerations.

Art psychotherapies have been increasingly recognised as a valid treatment for many mental health conditions, utilising all forms of artistic expression such as visual and performing arts and creative writing to facilitate expression and identity formation [28]. Research and theory into the application of art therapy for people living with BPD and other personality disorders has increased in recent years [28–30], with positive reports on acceptability of this approach from people with lived experience of personality disorder [31]. Recent studies have demonstrated benefits of art therapy for people diagnosed with personality disorder in a randomized controlled trial, with improvements in symptoms and increased self-regulation [32], and qualitative benefits in improving personal integration, emotion regulation, behaviour change and insight [33]. Recently, Haeyen, Chakhssi and Van Hooren [34] found that participation in art therapy improved areas such as emotional expression, self-image, sense of autonomy, insight into internal experiences (thoughts, feelings and behaviours) and managing vulnerabilities for people diagnosed with personality disorder over 3 months. This research has recently provided a platform for the development of an art therapy program focussing on building compassion for the self and others [35]. A further study of art therapy for people living with BPD suggests that the creative process facilitates improved mentalisation, by increasing flexibility to slow this process down to a manageable pace and anchoring mental content in an externalised format [36]. Research into the lived experience perspective of this approach identified improvements in resilience, mentalising and interpersonal safety, communication through art, and coping [37]. In this way, art therapy has been suggested to improve the challenges of living with BPD by embedding coping skills and understanding of the self and others. However, there are no studies to date evaluating a peer with lived experience leading an art therapy program.

People living with BPD report a desire for both helpful coping and survival strategies, and improved capacity to engage in meaningful activities and relationships in building their life worth living [22, 38, 39]. Recent advances in the treatment of mental health conditions includes the integration of lived experience peer workers as facilitating a holistic, compassionate trauma-responsive framework. A recent study examined the qualitative perspective of people living with personality disorder, those with lived experience of supporting them, and the integration of peer workers to the treatment of BPD [11]. The results indicate that the use of peer workers with lived experience of BPD can provide hope, connection, and validation to people living with BPD. Peer workers with similar lived experience have a unique capacity to provide an authentic understanding, role model personal use of therapeutic skills and strategies, and increase accessibility by reducing the power imbalance. In particular, the Intentional Peer Support (IPS) model [40], utilises a trauma and diversity-informed structured process outside of the medical model of care for developing equitable relationships to facilitate purposeful mutual learning and intentional change [41, 42]. IPS provides the opportunity to co-create new ways of thinking and responding, developing new self-narratives, and personal growth through meaningful peer relationships. IPS is founded on principles of ‘learning and growing, caring for relationship and hope’, which are explored through ‘connection, worldview, mutuality and moving toward’. It is therefore likely that prioritising peer design and facilitation in programs for BPD will enhance the experience of people with lived experience and allow them to move towards a life worth living.

The global coronavirus pandemic (Covid-19; declared 11 March 2020), has significantly impacted access to mental health services and increased psychosocial stress [43, 44], in particular for various vulnerable groups such as those with pre-existing mental health conditions (e.g., [43]). During a time whereby non-essential hospital use was discouraged, personality disorder related presentations were seen to increase, compared to decreases in other mental health groups such as psychotic and mood-related disorders [45]. Whilst this data is preliminary, it is possible that increased presentations reflect the complex interaction between the particular difficulties of people with personality disorder when faced with the unprecedented stress of a global pandemic including social isolation, financial concern, health anxiety and reduced access to usual services and supports [46]. However, the pandemic has created an opportunity for online programs and services to be developed and evaluated. Art therapy has been increasingly delivered online, resulting in improved accessibility and inclusivity [47]. Emerging literature suggests positive engagement and effectiveness of online art therapy with people experiencing mental health difficulties [48, 49]. Mental health programs have also emerged in the online format, including for people living with BPD [50, 51], and have been suggested to mitigate some of the adverse impacts of social restrictions [52]. However, there are currently no studies evaluating an online art therapy group for people with personality disorder.

The present pilot study evaluated an online arts-based skills program for people living with BPD, developed and led by a peer mental health professional. The evaluation was designed to assess the acceptability of the program, its role in ameliorating symptoms, and to evaluate if it improved emotion regulation.

## Methods

### The program

The program involved one 2-hour session every week for 18 weeks. The program was designed to be delivered on a secure online platform due to the global pandemic (Covid-19). Examples of practical adaptations for an online environment included providing access to art materials and workbooks to participants via mail delivery, consideration regarding positioning of the camera on the facilitator and participants and their artmaking. Clinical considerations included managing group process in the online space by providing opportunities for sharing and connection in diverse ways (such as non-verbal communication) and considering safety and management of potential risk. Online delivery of the program allowed broad engagement across many locations and within populations with increased vulnerabilities and considerations such as mobility and access issues.

Minimal contact with the facilitator was negotiated outside of the sessions, and connection was promoted through between-session art sharing via email or on a private social media platform. The groups were closed and capped at 10 participants. The pilot included 4 programs running concurrently from July to December 2020. Participants were able to self-select the program that best suited their schedule.

**Image 1:**
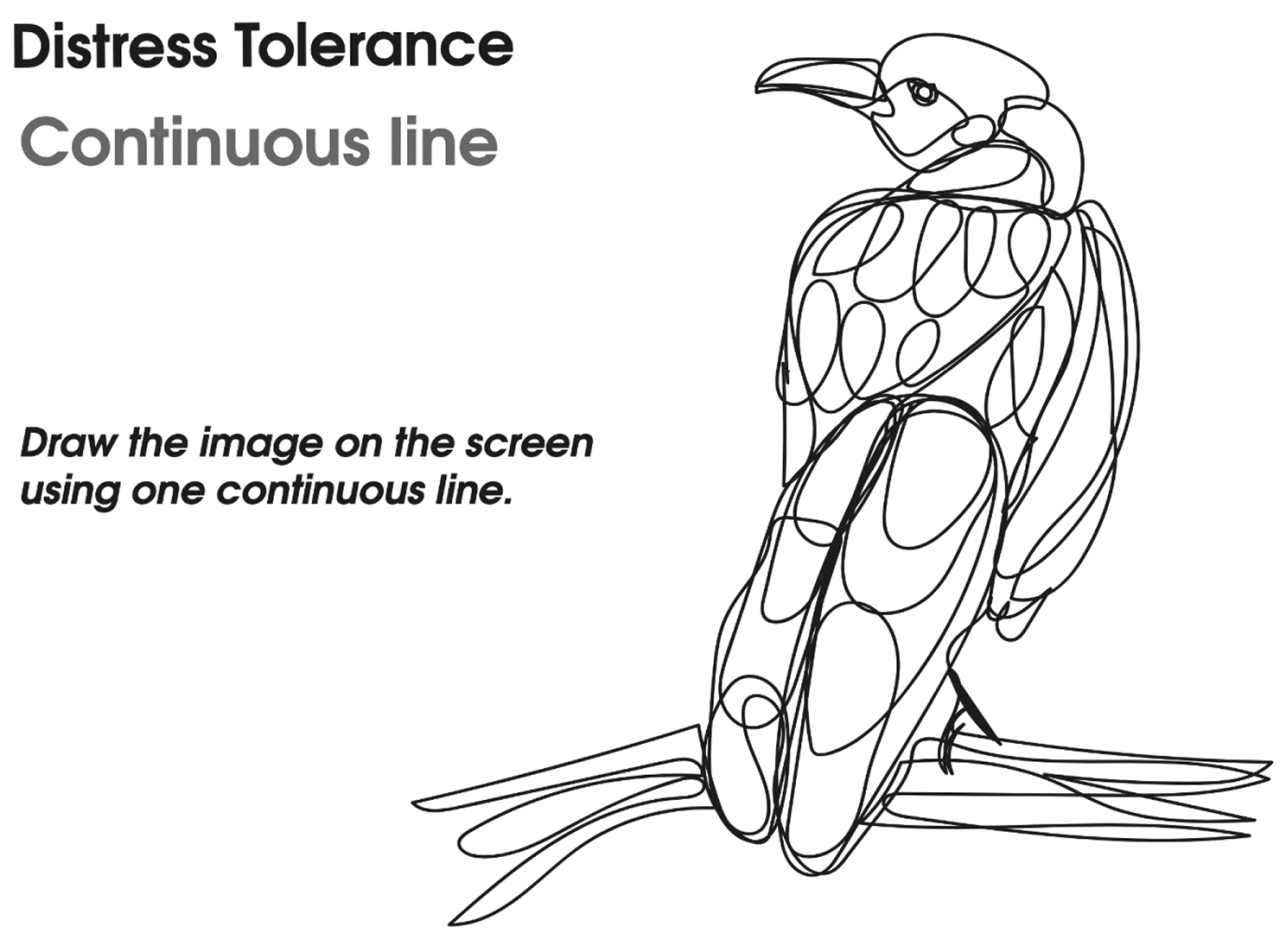
Example artmaking activity template for distress tolerance (continuous line). Jewell, M. “distress tolerance - continuous line”, 2020, Wangal Land

**Image 2:**
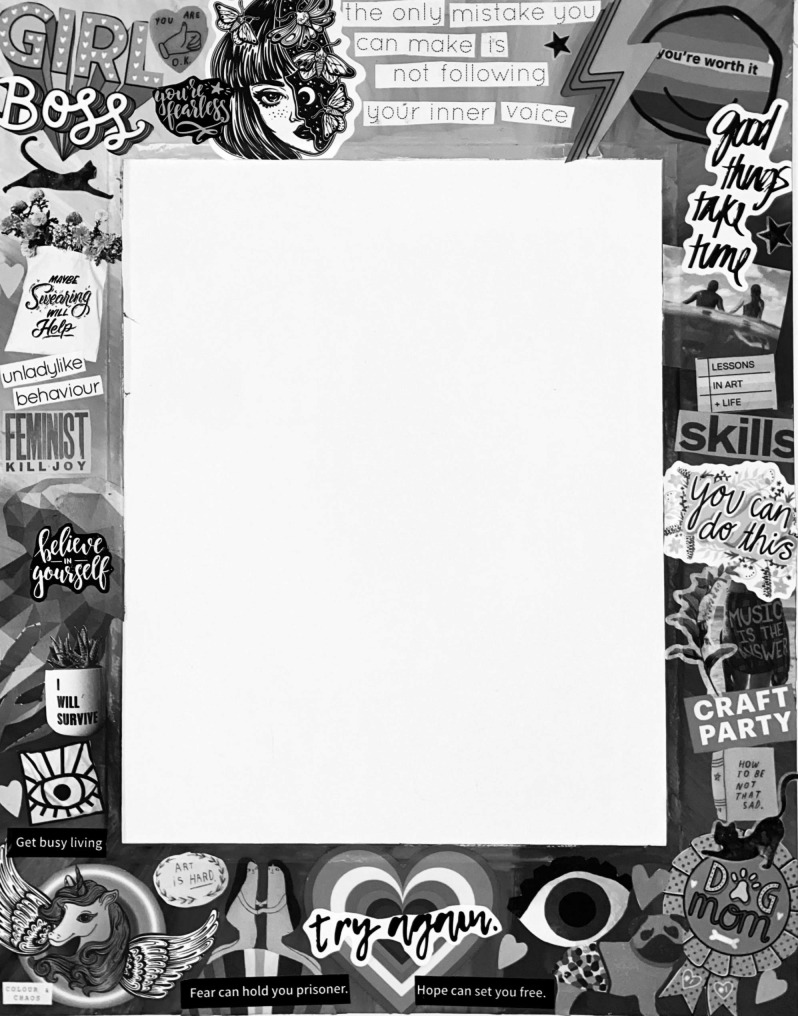
Example artmaking activity template for identity work. Task is to make a frame similar to this around an image or mirror, containing positive self-affirmations. Jewell, M. “the self frame”, 2020, Wangal Land

The program included skills based on dialectical principles and also specialist topics on self-stigma, invalidation and anger. See Table 1 for an outline of the 18 week program. Participants were provided with a booklet containing skill explanations and task templates, weekly pre-session emails regarding the focus and materials required for the session, and post-session recap emails with further resources. At-home art kits were also supplied to the participants. Artmaking activities were linked to skill-based learning during each session. See Image 1 and 2 for example artmaking activity templates provided.

**Table 1:** Outline of the 18 week program

Week	Topic	Content	Example Art Activities
1–4	Distress tolerance	Practical skills and tasks to support tolerating distress in real time.	Repetitive Shape and Line Continuous Line Self-care starters
5–8	Emotion regulation	Learning to label and connect with emotions to identify vulnerabilities. Skills to practice pre-emptive strategies to reduce the effects of emotional dysregulation.	Colouring emotions Positive memory bank Hidden emotions
9–12	Identity	Building connection to the self by identifying key value systems, processing and harnessing anger, validating the effects of external stigma and identifying self-stigma	Visualising values Motivational anger Flipping the script The self frame
13–16	Interpersonal Effectiveness	Developing skills in creating and asserting boundaries, understanding and navigating relationships using validating and active communication techniques	DEARMAN cards The story of us Staying Connected
17–18	Mindfulness	Skills and practices that encourage engaged participation outside of traditional mindfulness practices that are trauma informed, non-colonising and respectful of diversity.	Here and now Connecting to Country The growing tree

The 2-hour sessions included an Acknowledgement of Country and brief welcome with topic review (totalling approximately 15 min). The session then moved towards providing information on the first skill (15 min) with artmaking time (20 min) and participant reflection and feedback (20 min). This process was repeated for the second skill of the session, with a brief 5-minute conclusion. Homework tasks were incorporated prior to the first and last sessions to enhance engagement and reinforce learning.

### The facilitator

The lead author (MJ) designed and conducted the program, and the third author (RC) was a peer model/support facilitator. The lead author has Dialectical Behaviour Therapy, counselling, Narrative Therapy and art psychotherapy training and lived experience of complex mental health including BPD. They are a registered Art Psychotherapist, hold a Masters of Art Therapy, and are an Intentional Peer Support Practitioner. The peer facilitation was grounded by the IPS model of practice and a trauma and diversity informed framework. No fidelity assessments were utilised.

The primary role of the psychotherapist facilitator was to provide a safe and inclusive therapeutic environment, that was sensitive to group and individual processing needs. They used the arts-based program to experientially explain and practice skills. Sharing and self-expression in a safe and trauma-informed manner was encouraged, including managing any individual considerations (e.g., symptom elevation or risk), whilst maintaining the frame of the group (e.g., time management, active participation, etc.). The psychotherapist facilitator demonstrated skilled behaviours, modelled effective boundaries, and provided considered and therapeutically-oriented self-disclosure of lived experience where appropriate. The lived experience facilitation was demonstrated by providing personal artwork examples and using personal narratives connected to the overall topic and skills being discussed. Replication of this intervention would require an Art Therapist with lived experience, which could be sourced using programs such as the BAAT “Dual therapist” program or with a peer worker co-facilitator. It is essential that the facilitator conducts the program within both the DBT and IPS framework. This includes maintaining a non-judgmental approach, being open to the experience of the participants, actively engaging in co-creating, meaning-making and mutuality, dialectically recognising that there is no absolute truth, and maintaining a hopeful stance.

### Participants

The program was advertised by contacting personality disorder services, wider mental health services, advocacy groups and research centres across Australia. The program was evaluated on people with lived experience of Borderline Personality Disorder. Participants were required to have received a diagnosis of Borderline Personality Disorder from a psychiatrist, however diagnoses were not repeated by the research team. Most participants had some prior treatment experience. Participation in the program was paused if they became acutely unwell or suicidal and required urgent mental health care. Participants were considered to have dropped-out of the program if they did not attend three sessions in a row or complete the final two sessions. All participants provided informed consent to participate in the pilot program, including the collection of evaluation data. The art therapy program was approved by the Institutional Review Board (University of Wollongong HREC#220).

38 participants enrolled in the program. 89.5% of participants identified with she/her pronouns, and 10.5% identified with they/them pronouns. Participants reported a mean age of 33.6 (range 18 to 54 years, SD = 9.58). Most participants resided in metro regions across Australia (57.9%), with the remaining living in regional or remote areas of Australia (28.9%) or internationally (13.2%). International participants were located in New Zealand, the United Kingdom and United States.

All participants were living with Borderline Personality Disorder. Participants also reported comorbidities including anxiety disorders (68.4%), post-traumatic stress disorder (44.7%), depressive disorders (55.3%), eating disorders (26.3%), bipolar disorders (15.8%), attention deficit hyperactivity disorder (5.3%), autism spectrum disorder (5.3%), adjustment disorder (2.6%), psychosis (2.6%), and chronic physical health conditions (15.8%).

Prior experience with therapy varied. Most participants had previously completed a therapy program (39.5%), started but not completed a therapy program (18.4%), or was either currently engaged in a therapy program or on a waiting list (13.2%). Some participants stated that a therapy program wasn’t available to them (13.2%). 68.4% of the participants reported that they regularly attend sessions with a mental health professional, and 50% stated that they often rely on the support of family members or friends.

We tested whether ongoing engagement with a mental health professional at baseline impacted DERS scores at pre and post-program. There was no significant difference on the baseline DERS total score between those who reported regularly engaging with a mental health professional (M = 109.65, SE = 5.02, SD = 25.58) compared to those who were not regularly engaged (M = 121.33, SE = 6.65, SD = 23.04), t [36] = -1.35, p = .186. At post-program this lack of difference remained between groups (M = 89.25, SE = 8.16, SD = 23.07), t [29] = − 0.705, p = .486. In addition, a one-way ANCOVA was conducted to determine whether a statistically significant difference occurred between participants who reported regularly engaging with a mental health professional compared to those who were not on the post-program DERS controlling for the pre-program DERS as a covariate. There was no significant effect of engagement with a mental health professional on the post-program DERS after controlling for the pre-program DERS, F [1, 28] = 0.17, p = .686.

Prior to the program, participants reported variable engagement with artmaking, where 31.6% stated that they usually spent less than 1 h a week artmaking pre-program, 28.9% 1 to 2 h a week, 23.7% 2 to 3 h per week, and 15.8% over 4 h per week.

### Measures

Participants completed a brief questionnaire prior to commencing the program (baseline), at the mid-point (9 weeks) and at completion (18 weeks). Measures included both quantitative and qualitative approaches to assess acceptability of the program, and pilot data regarding effectiveness on symptom improvement.

### Difficulties in emotion regulation scale (DERS)

The DERS [53] is a 36-item scale measuring non-acceptance of emotional responses, difficulties engaging in goal-directed behaviour, impulse control, emotional awareness, access to emotion regulation strategies, and emotional clarity. Higher scores represent greater difficulties in emotional regulation. The total score at baseline resulted in strong internal consistency (α = 0.95, N = 38). The DERS is a standard outcome measure in BPD research, particularly approaches using DBT.

### Acceptability

Acceptability is an important measure of participant experience and satisfaction of a pilot program and has implications for future replications. Acceptability is often measured through a combination of simple quantitative scales, and qualitative feedback. This method has previously been utilised by pilot programs in the treatment of people with BPD (e.g., [54, 55]). In the current study, program acceptability was assessed through online survey evaluation questions at completion. Participants were able to endorse statements reflecting their experience over the duration of the program. Examples included whether the participant would be more likely to engage in therapy in the future if they contained art-based skills, whether the facilitator having lived experience influenced the participant’s decision to complete the program, and whether the program improved participants social interaction and coping skills. Acceptability of the program was also supplemented by the qualitative feedback provided by participants.

### Qualitative questions

The post-program evaluation also included free-text qualitative questions including ‘*How has the group affected your understanding of therapeutic skills and theory?*’, ‘*How has the group affected your sense of self?*’, and ‘*How has the group affected the way you view “art as healing” and/or “art as therapy”?’*. The qualitative data provides participant feedback on satisfaction, experience and acceptability of the program.

### Statistical analysis

The pre and post-program main outcome (DERS) was assessed using a multilevel modelling approach (IBM SPSS Statistics 26 Linear Mixed Models). Utilisation of this statistical approach allowed for analysis of all data as intention-to-treat [56], with time as a repeated measure and DERS as the dependent variable. The covariance structure for the residuals was specified as antedependent (first order), which is often utilised in repeated measures data [57]. Unpaired t-tests were used to compare the means of the current sample on the main variable of interest (the DERS) to the means previously reported in the literature. This analysis provides further insight into the symptomatology of the current sample at baseline, and allows comparison to pseudo-control groups at pre and post-program.

Qualitative data was analysed using a Husserlian phenomenological approach, previously described and applied in qualitative data analysis [58]. This approach aims to derive meaning of the lived experience by asking broad questions, ‘bracketing’ the researcher’s preconceptions, and extracting themes. Researchers became immersed in the data prior to analysis. Significant statements relating directly to the phenomenon under study were then extracted. Approximately 20% of the full qualitative dataset was coded into themes by two researchers independently, and discrepancies were discussed until consensus was reached. Both coding researchers were independent of the development and facilitation of the program to reduce potential bias. Inter-rater reliability was assessed with Cohen’s kappa coefficient being K = 0.72, indicating a good level of agreement.

## Results

### Attrition

31 participants completed the program (N = 7, 18% drop-out rate). There was no significant difference on the baseline DERS total score between those who completed (M = 112.97, SE = 4.82, SD = 26.85) compared to those who did not complete the program (M = 115.00, SE = 6.29, SD = 16.63), *t* [36] = − 0.19, *p* = .85. In addition, there was no significant demographic difference between those who completed compared to those who did not complete based on identified preferred pronoun *X*^2^ [1] = 1.009, *p* = 1.00 (adjusted by Fisher’s Exact Test), or age *t* [36] = 0.004, *p* = .997.

### Main outcomes

On the main outcome variable, DERS, the baseline mean was 113.34 (SD = 25.10). This was compared to comparison groups in the literature using this measure. The current average baseline DERS was lower than previously reported by research with people diagnosed with BPD ([59]; M = 121.37, SD = 22.07, N = 138), *t* (417) = 3.803, *p* = .035. It is likely that this is due to most of the sample having already completed, or currently engaged in, a therapeutic program.

The DERS was also compared to pseudo-control groups in the literature. The current sample reported significantly higher DERS at baseline than previously reported by male and female university students ([53]; M = 79.33, SD = 19.76, N = 357), *t* (393) = 9.80, *p* < .0001, and female controls ([60]; M = 67.95, SD = 14.46, N = 20), *t* [56] = 7.44, *p* < .0001. This suggests that the group was symptomatic in experiencing difficulties with regulating their emotions prior to the program.

The post-program mean on the DERS was 84.52 (N = 31, SD = 21.85). This remained significantly higher than previously reported by female controls (as above; [60]), *t* [49] = 2.99, *p* = .004. However, the post-program DERS was no longer significantly different to male and female university students (as above; [53]), *t* (386) = 1.39, *p* = .17. Therefore, the variable of interest showed improvement at post-program when compared to a university sample pseudo-control group previously reported in the literature.

**Fig. 1:**
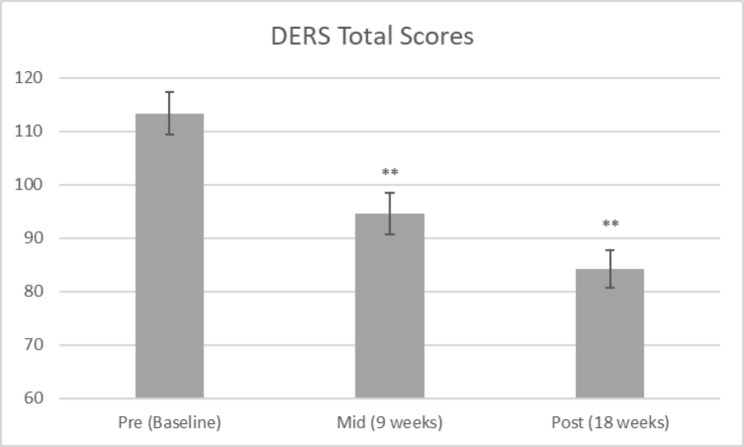
Difficulties in Emotion Regulation Scale (DERS) total score across time points. ** denotes significance at p < .01 (Pre > Mid, Pre > Post, Mid > Post)

Linear mixed model analyses demonstrated a significant reduction in the DERS total score over time, *F* (2, 53.34) = 34.05, *p* = .000. Figure 1 depicts the significant reduction in difficulties regulating emotions at the three time points. This demonstrates a significant improvement in emotion regulation following the program. Comparison of the DERS means from pre to post-program results in *Cohen’s d* = 1.77, indicating a very large effect size.

**Fig. 2:**
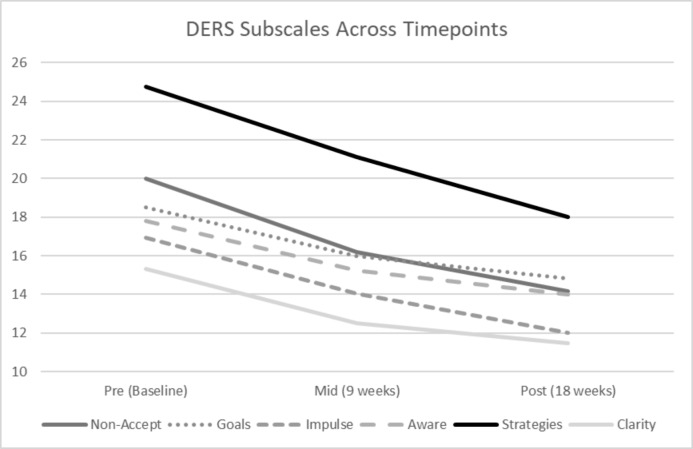
Average Difficulties in Emotion Regulation Scale (DERS) subscale scores across time points. Non-accept = non-acceptance of emotional responses; Goals = difficulties in goal-directed behaviour; Impulse = impulse control difficulties; Aware = lack of emotional awareness; Strategies = limited access to emotion regulation strategies; Clarity = lack of emotional clarity

To further elucidate the significant improvement of DERS over time, linear mixed model analyses were performed on all the subscales. All subscales demonstrated a significant improvement over time; non-acceptance of emotional responses, *F* (2, 42.42) = 16.64, *p* = .000; goal-directed behaviour, *F* (2, 44.15) = 17.26, *p* = .000; impulse control, *F* (2, 47.79) = 20.10, *p* = .000; emotional awareness, *F* (2, 61.13) = 9.08, *p* = .000; access to emotion regulation strategies, *F* (2, 51.90) = 19.49, *p* = .000; and emotional clarity, *F* (2, 55.55) = 14.96, *p* = .000. Figure 2 depicts the change in DERS subscales over time. This indicates significant improvement across all domains of emotion regulation at post-program.

### Acceptability

The lived experience facilitation of the program was important to participant’s engagement. From the outset, 71.1% endorsed that the peer facilitation made it more likely for them to participate, and 10.5% stated that this was the only reason they registered. At completion, 100% of participants stated that the facilitator having lived experience positively impacted on their decision to stay in the program. In addition, 100% of participants endorsed that their experience in the current program would positively impact on their decision to engage in further therapy or support interventions in the future if they contained art-based skills.

At completion, 93.5% of participant’s stated that the program had increased their understanding of therapeutic skills and principles, and 93.5% endorsed that the program had helped participants to implement skills in ways that they hadn’t been able to before. Additionally, 80.6% stated that the program supported them to stay living well, and 77.4% that the program had increased their wellbeing. 90.3% endorsed that the program helped them understand themselves (including their thoughts, feelings and behaviours), 80.6% increased self-compassion and self-worth, and 83.9% that the program allowed the participants feel seen, heard and validated. 61.3% stated that the program increased their social interaction skills, and 87.1% increased their confidence in using art as a tool for healing. Finally, 67.7% stated that the program added benefit to the verbal therapies the participant was engaged in, and 67.7% that the program provided peer support that they had never had access to before. Taken together, these results indicate high acceptability of the program. Acceptability of the program was further elucidated through the qualitative feedback.

### Qualitative outcomes

Thematic analysis of the qualitative data revealed many significant statements regarding the impact of the program on meaningful change for people living with BPD. Table 2 provides examples of significant statements with the formulated meanings.

**Table 2:** Selected examples of significant statements of participants experience of the program and formulated meanings

Significant Statement	Formulated Meaning
1. **Improved capacity to regulate emotions and tolerate distress**	
a. “Learning the… skills using art as a tool has made a massive impact on me… I have been able to retain and utilise skills in ways I haven’t in the past.“ “Understanding [the skills] from an artistic approach has significantly increased my awareness and my willingness to sit with my feelings and use the skills.”	a. Participants reported improved accessibility and practical application of the therapy skills
b. “I’ve since used art on several occasions instead of reverting to one of my less helpful coping strategies, and I look forward to doing so more in the future. I honestly think it may even have saved my life on one of the occasions I utilised it.”	b. Participants reported that art is a useful coping strategy
c. “I have been able to embrace the process over product more and create for the sake of creating, create just because it feels good.”	c. Participants reported increased positive experiences through enjoyment of art
2. **Connection with others**	
a. “The peer facilitation has also greatly improved the way I view the skills, the situations in which I would benefit from being skilful and my willingness to be skilful in general.”	a. Participants valued the lived experience perspective of the facilitator
b. “I have also gained confidence and know that I can be accepted and supported and can show vulnerability without feeling so uncomfortable. These are things I’ve always found challenging however I have been given an opportunity to learn in a supportive group.”	b. Participants benefitted from the supportive group process
3. **Improved understanding of the self**	
a. “The group has improved my sense of self by helping me to look at my thoughts, feelings and behaviours and to explore my strengths and values.” “I definitely feel like I understand myself better though, especially in terms of my feelings.”	a. Participants felt more connected to their internal experience
b. “… [Art] allowed me to express my emotions and feelings in an easier and more effective way.” “I am now using art to connect with people, to communicate with people, and to heal my own soul.”	b. Participants reported improved self-expression
c. “I believe art is a great way in to ourselves and can assist with healing and processing of events.”	c. Participants reported therapeutic benefits of artmaking
4. **Hope for living well**	
a. “It has given me a lot of hope for my own recovery in BPD and other mental health challenges.”	a. Participants felt more hopeful for their capacity to live well
b. “I feel like I deserve to own my identity, that I am not just a sick person, and that I deserve to ask for what I need.”	b. Participants felt empowered

### Theme 1: improved capacity to regulate emotions and tolerate distress

Participants reported improved understanding of therapeutic skills and principles through artistic practice. The practical nature of the skills was a common theme, facilitating improved accessibility and meaningfulness of otherwise abstract or theoretical concepts. This, in turn, enhanced practical application, increased capacity for coping and regulating emotions, and willingness to try new strategies. One person living with BPD stated:

“It’s made a few skills easier to understand, and making them more interactive, makes me want to practice them more often.”

Another person living with BPD commented on improved understanding of the integration of therapeutic skills, and understanding of the scientific and theoretical underpinnings:

“I was able to learn about some of the theories being explored through the activities. The activities and artworks created in each session encompassed multiple… skills which showed how they were all interlinked rather than separate skills to be used one at a time. While the activities aren’t necessarily strategies to take with me ‘on-the-go’ (although some are) they do help in opening me up to a more realistic understanding of [the skills].”

Participants also expressed the benefits of enjoying the process of artmaking. Many participants reported that this process facilitated the increase of positive experiences, thus increasing their capacity to cope and engage in self-care.

### Theme 2: connection with others

Participants expressed the benefits of engaging in a supportive group process, including practicing being vulnerable and connecting with others. Some participants commented that this was a challenging process that also allowed them to practice being skilful. Participants also reported the value of the peer-facilitation in providing lived experience perspectives, and authenticity to the process and skills. It was acknowledged that lived experience examples of how skills could be applied was useful to increasing understanding and willingness to try new skills. Participants reported that the lived experience also facilitated validation beyond what non-peer mental health clinicians can provide. One participant said:

*“It was made easier to understand by being facilitated by someone with lived experience and I felt more validated, understood, and more comfortable engaging. This was not only more accessible for me but it was delivered in a way that kept me coming back each week.”*

### Theme 3: improved understanding of the self

Improvements in understanding of the self were reported by participants, including greater awareness of internal processes. In particular, participants reported greater connection to their thoughts and feelings. Participants commented that the program facilitated better self-expression of their internal world. Many participants reported sharing their artwork with others involved in their support systems, providing a platform to facilitate deeper understanding and discussion. In addition, participants reported the therapeutic value of the program. Many participants reported experiencing a strengthened self-concept, including a deeper appreciation of their own values and beliefs. Some participants also reported the benefits of being able to process important emotions and events to facilitate wellbeing.

*“Now I know that all my feelings are valid, I can accept myself as a whole being rather than feeling “split” between good and bad. When I feel upset, I know how to self-soothe and validate and then I move on to check the facts and problem-solve. I really feel that I accept myself more as a person - it’s been many years in progress, but this group has helped me to stop feeling shame and hatred towards myself, as I now understand how to “look after” myself. I feel so much more stable than I ever have.”*

Another person living with BPD commented:

“I used to say I had no genuine sense of self and that I would change myself to please others. Since then I have learnt about boundaries, the importance of values and how we can create our own story by reclaiming the hurtful and invalidating things we are told by society. While I’m not yet a completely self-assured individual, I definitely have more of an idea of ‘Me’ than 18 weeks ago.”

### Theme 4: hope for living well

Participants expressed a greater sense of hope for their own ability to live well following the program. This was often linked to connecting with a peer mental health professional and witnessing the possibility of living well during this process. Many participants also found a deeper understanding of the self and capacity to regulate as instilling hope for a life worth living. In addition, many participants also reported an increased sense of empowerment following the program. One participant powerfully stated:

“I feel much more confident in myself and my decisions. I feel more valuable and like my feelings and thoughts matter.”

## Discussion

The present study evaluated a peer-led art-based skills program for people living with BPD. A multilevel modelling analysis of the primary outcome variable (DERS) demonstrated significantly improved emotion regulation over time, with a large effect size. Significant improvement was observed across all subscale domains of this measure, indicating wholistic improvements in emotion regulation over the course of the art therapy program, and thus reduced symptomatology of Borderline Personality Disorder.

Taken together, results show that participants found the approach to be acceptable, and potentially effective. Acceptability of the program is further supported by the strong retention (82%). This is compared to 67% retention in a study of mentalisation-based treatment program with an art therapy component for people with lived experience of BPD [36], 77% in a randomised controlled trial of art therapy for people with personality disorder [32], and 73% retention in a recent study evaluating a standard DBT skills only group intervention for people with lived experience of BPD [18]. Qualitative analysis of participant feedback found key themes indicating improved capacity to regulate emotions and tolerate distress, improved connection with others, enhanced understanding of the self, and hope for living well. Within these themes, subthemes emerged indicating improved accessibility and practical application of therapeutic skills, appreciation of the peer-facilitation and supportive group process, enhanced self-concept and empowerment. Taken together, these results suggest promising benefits of the program in providing a platform for deeper self-understanding and meaningful change.

Many participants commented on the benefits of the peer facilitation aspect of the program. Often it was explained that this provided an authentic space to understand the skills, increasing practical application and willingness. Whilst the mechanism of symptom change in this program is currently unknown (the peer-facilitation and peer-support, the skills, or the art psychotherapy approach), the IPS model of mutuality in meaningful relationships appeared important to the participants. This is consistent with previous research on the perceptions of people living with BPD, where peer workers were described as being a role model of the use of skills, and providing the opportunity for genuine validation with similar lived experience [42]. This study therefore supports the use of peer workers and peer mental health professionals in the treatment of BPD, in particular within the structured framework of a group-based approach.

The artmaking component of the program was also highly valued. Participants reported improved capacity to both understand and express themselves through the process of artmaking. In addition, participants reported greater applicability and integration of the skills. People living with BPD reported a stronger sense of their own values, strengths and internal processes, describing the capacity for artmaking to facilitate the process of developing self-concept and identity. Whilst traditional evidence-based treatments for BPD show significant benefits in acute symptom improvement (e.g. reduced suicidal and self-harm behaviours in DBT; [61]), it is possible that art therapy programs may compliment and extend on these by addressing one of the core challenges inherent in personality disorders (complex identity; [62]). The capacity for arts-based practice to facilitate deeper self-exploration and identity development may provide additional benefits to people living with BPD in reducing feelings of emptiness and therefore risk of acute symptom re-emergence [23]. Indeed, people living with BPD report the importance of developing meaningful relationships, and strengthening a sense of self [63]. Providing choice in treatment options contributes to developing the persons growing sense of agency, which is core to the treatment of personality disorder [64], and is recommended by current treatment guidelines [4, 6, 28, 65, 66]. It is also possible that art psychotherapies may increase engagement and accessibility of therapy for people with lived experience who would otherwise find the traditional therapeutic environment too colonised, conceptualised, medicalised, overwhelming or exclusive given their particular needs. This may include those who are culturally and linguistically diverse, non-verbal, neurodiverse and live with co-occurring chronic illness and disability. Therefore, art therapy may be a beneficial counterpart to traditional evidence-based psychotherapy treatment programs for BPD, or an alternative pathway for people who have challenges accessing traditional therapeutic approaches.

The online format of the program may have also contributed to an inclusive treatment pathway for people who have challenges accessing traditional therapeutic approaches. The current program was designed for the online format due to the global pandemic. Accessibility was improved using the online modality, including those due to Covid-related social restrictions, geographical considerations, and the intrinsic interpersonal difficulties associated with the disorder. The Relational Model suggests that the underlying difficulty of BPD stems from problematic relationships developed with the self and other over time [7]. As such, engaging in treatment programs in either a group or individual format may be experienced as particularly threatening or overwhelming for people with BPD, with core difficulties in interpersonal relating. These factors are well understood and personally experienced by the facilitator and drew on the expertise of lived experience. Research into the experience of online therapy in other population groups has highlighted the potential benefit of increased access, convenience, and reduced anxiety regarding some of the interpersonal aspects of therapy. However, disadvantages may include disconnection from the therapeutic environment, warranting the need for creativity and experience in delivering the intervention and facilitating rapport (e.g., [67]). It is therefore possible that online therapeutic programs may be of similar benefit to people with personality disorder by titrating the intensity of the interpersonal therapeutic space to focus on an initial acquisition of emotion regulation skills in a creative way. In this way, the person may experience a positive introduction to therapy, and may be more likely to further engage in therapeutic approaches. Future research may therefore benefit from further understanding the experience of people with lived experience of BPD regarding the online therapeutic format, including treatment preference and alternative pathways.

The present study has limitations. The small sample size limits the generalisability of the findings. The pilot involved an uncontrolled pre-post evaluation design, and therefore further research is required to replicate and extend on these results to increase confidence in the benefits of the program, and whether these are maintained over time. In order to maximise inclusivity and respect the diagnosis that participants self-identify with to describe their experience, diagnosis of BPD or other comorbid conditions were not formally assessed by the researchers involved in this study. Future research may consider integrating formal assessment procedures to confirm the diagnostic profile of participants. This may facilitate greater understanding of whether the program assists people living with BPD more than other diagnostic or comorbid groups. Of interest, this information may also assist to understand how people with emotion dysregulation understand and self-identify with their difficulties, and the therapeutic preferences of people with lived experience (e.g. art therapy compared to traditional psychological therapy approaches). In addition, the program was delivered online in the context of the Covid-19 global pandemic, which allowed participation from regional and remote areas, and international locations, who otherwise would not have been able to attend the program. However, whilst other art therapy programs have demonstrated successful implementation using telehealth (e.g., [68]), it is currently unclear whether changing this format to in-person would impact on the results. Approximately 50% of the participants engaged in artmaking for 1 to 3 h per week prior to the program, and therefore it is unclear whether prior attitudes towards art or art therapy impacted the recruitment, evaluation, and acceptability of the program. In addition, primary language information was not collected as part of the research design. Future online art therapy studies may benefit from understanding this demographic considering the potential for international participation. The pandemic-related social restrictions and stressors may have also influenced the efficacy of the program, and therefore suggests the need for further replication. Future studies may also benefit from understanding the peer facilitators experience of the program content, delivery and therapeutic process. Despite these limitations, this study shows promise in the benefits of an online peer-led art-based skills program in both participant acceptability, qualitative feedback and symptom reduction.

## Conclusion

The present pilot study evaluated a peer-led art-based skills program for people living with Borderline Personality Disorder. The results are promising, demonstrating strong acceptability of the program, positive and meaningful qualitative feedback, and large improvements in emotion regulation across time. The use of peer facilitation founded in the principles of Intentional Peer Support was found to be valuable in providing authentic validation and promoting willingness and hope for living well. In addition, the arts-based approach, informed by the dialectical framework and skills, was beneficial in promoting accessibility of the strategies and development of a sense of self. We found evidence that art therapy facilitated by a peer with lived experience assisted in the recovery of people living with Borderline Personality Disorder.

## Data Availability

Participants gave researchers consent to use the data for this evaluation, but not for further distribution outside of the research team.

## List of abbreviations

BPD: Borderline Personality Disorder
DBT: Dialectical Behaviour Therapy
IPS: Intentional Peer Support
